# Horse-Back Riding and Pulmonary Calisthenics

**Published:** 1884-03

**Authors:** 


### Horse-Back Riding and Pulmonary Calisthenics.

Dr. Dio Lewis writes a story in his new journal tending to illustrate the great benefits that consumptives will derive from horse-back riding. Whether this benefit is due to anything more than the necessity which it entails of being in the fresh air we do not know, but we do know that fresh air has remarkable influence on weak lungs, so-called. Whether the oxygen destroys the famous bacilli, or what is the modus operandi, we leave for others to determine, but we will recommend a little procedure that we have repeatedly found to make strong men and women out of those whom others as well as ourselves had pronounced to be afflicted with incipient phthisis. Throwing back the shoulders, we direct our patients to inspire and expire as forcibly and deeply as possible, expanding and contracting the chest to its greatest possibilities, and being in the open air to go through this process as often as possible in the course of the day. Try this plan, and it will surely give satisfaction.

